# Preliminary *T_c_* Calculations for Iron-Based Superconductivity in NaFeAs, LiFeAs, FeSe and Nanostructured FeSe/SrTiO_3_ Superconductors

**DOI:** 10.3390/ma16134674

**Published:** 2023-06-28

**Authors:** Chi Ho Wong, Rolf Lortz

**Affiliations:** 1 Department of Physics, Hong Kong University of Science and Technology, Hong Kong, China; 2 Department of Industrial and Systems Engineering, The Hong Kong Polytechnic University, Hong Kong, China; 3 Research Institute for Advanced Manufacturing, The Hong Kong Polytechnic University, Hong Kong, China

**Keywords:** iron-based superconductivity

## Abstract

Many theoretical models of iron-based superconductors (IBSC) have been proposed, but the superconducting transition temperature (*T_c_*) calculations based on these models are usually missing. We have chosen two models of iron-based superconductors from the literature and computed the *T_c_* values accordingly; recently two models have been announced which suggest that the superconducting electron concentration involved in the pairing mechanism of iron-based superconductors may have been underestimated and that the antiferromagnetism and the induced *xy* potential may even have a dramatic amplification effect on electron–phonon coupling. We use bulk FeSe, LiFeAs and NaFeAs data to calculate the *T_c_* based on these models and test if the combined model can predict the superconducting transition temperature (*T_c_*) of the nanostructured FeSe monolayer well. To substantiate the recently announced *xy* potential in the literature, we create a two-channel model to separately superimpose the dynamics of the electron in the upper and lower tetrahedral plane. The results of our two-channel model support the literature data. While scientists are still searching for a universal DFT functional that can describe the pairing mechanism of all iron-based superconductors, we base our model on the ARPES data to propose an empirical combination of a DFT functional for revising the electron–phonon scattering matrix in the superconducting state, which ensures that all electrons involved in iron-based superconductivity are included in the computation. Our computational model takes into account this amplifying effect of antiferromagnetism and the correction of the electron–phonon scattering matrix, together with the abnormal soft out-of-plane lattice vibration of the layered structure. This allows us to calculate theoretical *T_c_* values of LiFeAs, NaFeAs and FeSe as a function of pressure that correspond reasonably well to the experimental values. More importantly, by taking into account the interfacial effect between an FeSe monolayer and its SrTiO_3_ substrate as an additional gain factor, our calculated *T_c_* value is up to 91 K and provides evidence that the strong *T_c_* enhancement recently observed in such monolayers with *T_c_* reaching 100 K may be contributed from the electrons within the ARPES range.

## 1. Introduction

The pairing mechanism of unconventional high-temperature superconductors (HTSCs) remains one of the greatest unsolved mysteries of physics. All unconventional superconductors, including cuprates [1,2] and iron-based HTSCs [3,4] but also heavy fermions [5] and organic superconductors [6], have in common that the superconducting phase occurs near a magnetic phase. Furthermore, their phase diagrams typically show at least one other form of electronic order, e.g., charge or orbital order [7,8], a pseudogap phase [2], stripe order [2] or nematic order [9]. The proximity of the magnetic phases naturally suggests the involvement of magnetism [10]. In most theoretical approaches, spin fluctuations play a leading role [11,12]. Alternative approaches consider, e.g., excitonic superconductivity [13,14], long-wavelength plasmonic charge fluctuations or orbital fluctuations [15,16,17].

It is generally assumed that the Cooper pairing in these superconductors cannot be described within a standard phonon-mediated scenario. However, this assumption is based only on the consideration of electron–phonon coupling on the Fermi surface only. The *T_c_* calculation based on the McMillan *T_c_* formula typically uses an approximation valid for classical low-*T_c_* superconductors, where the superconducting electron concentration is only considered at the Fermi level. This approximation is no longer valid for high-temperature superconductors such as the iron-based superconductors, since high-energy phonons are excited at elevated temperatures, so that electron–phonon scattering influences the electron over a larger energy range around the Fermi energy. In the high-temperature limit, this energy range may be comparable to Debye energy. Experimental ARPES data actually show that in iron-based superconductors, electrons down to ~0.03–0.3 eV below the Fermi energy are influenced by the onset of superconductivity [18,19,20]. In order to perform a comprehensive study of whether the electron–phonon coupling is related to the formation of Cooper pairs in iron-based superconductors or not, we decided to consider the true superconducting electron concentration in order to recalculate the electron–phonon coupling constant under an antiferromagnetic background. Several studies offered an alternative scenario for iron-based superconductors, suggesting that the role of electron–phonon coupling had previously been underestimated against antiferromagnetic (AF) backgrounds [21,22,23]. An explicit Density Functional Theory (DFT) calculation by B. Li et al. [22] showed that the phonon softening of AFeAs (A: Li or Na) under an AF background allows an increase in the electron–phonon coupling by a factor of ~2. While any orthogonal change in the phonon vector can be considered a phonon-softening phenomenon, the lattice dynamics studied by S. Deng et al. [23] confirmed that out-of-plane lattice vibration amplifies electron–phonon scattering based on their first-principle linear response calculation. While the tetrahedral atom is better suited to attract electrons in terms of electronegativity, the vertical displacement of the lattice Fe transfers the charge of the electron to the tetrahedral regions to generate an additional *xy* potential [21]. S. Coh et al. [21] calibrated the GGA + A functional, which made it possible to bring the simulation results much closer to the experiments [21,24]. The calibrated ab initio method explicitly demonstrates the occurrence of the induced *xy* potential from the out-of-plane lattice dynamics in the AF background that increase the electron–phonon scattering matrix by this factor of ~2 (abbreviated as ratio *R_ph_*). More importantly, they provide an analytical model [21] to explain why the electron–phonon scattering computed by the ab initio method is always increased by a ratio of ~2 under the effect of the spin density wave (abbreviated as ratio R_SDW_).

The pairing strength of iron-based superconductivity can be enhanced significantly with the help of nanostructuring [22,25,26,27]. The layer structure of FeSe makes it possible to grow monolayers of FeSe epitaxially on a substrate. In 2013, superconductivity was reported with a record *T_c_* of 70 K on monolayer FeSe on a SrTiO_3_ substrate [25], which was later increased to 100 K [26]. Despite the complexity of the electronic phase diagram of iron-based superconductors, which suggests the presence of additional broken symmetries besides the broken U(1) gauge symmetry of the superconducting state and thus an unconventional pairing mechanism, recent works have suggested that electron–phonon coupling could play a certain role in the superconducting mechanism of iron-based superconductors [22,27,28], although there is clear evidence that magnetic fluctuations must be taken into account. The high transition temperature of the monolayer FeSe on a SrTiO_3_ substrate gives further indications of the importance of electron–phonon coupling. While growing FeSe films on graphene substrate suppresses *T*_c_ [29], the giant enhancement of *T*_c_ is likely activated by the SrTiO_3_ substrate, where the interfacial contribution cannot be ignored. Strong electron–phonon coupling at the interface of FeSe/SrTiO_3_ has been identified in ARPES data [19], with electrons located 0.1–0.3 eV below the Fermi level involved in superconductivity. Although the FeSe phonons do not depend on the thickness of the FeSe material, unusual phonons [30,31], such as the F-K phonon, across the interface may be responsible for the high *T*_c_ [31]. According to the experiment by S. Zhang et al. [31], the F-K phonons of the FeSe/SrTiO_3_ surface show new energy loss modes, and the line width is widened compared with bare SrTiO_3_.

In this article, we revise the superconducting electron concentration and use an ab initio approach to examine if the *T_c_* values of LiFeAs, NaFeAs and FeSe as a function of pressure can be calculated reasonably by taking into account the R_ph_ and R_SDW_ factors, etc. If successful, we use this model to test whether such an approach can be applied to the ~100 K superconductivity in the nanostructured FeSe/SrTiO_3_. Not all mechanisms of iron-based superconductivity have been encountered in this work, because the unified theory of iron-based superconductors remains an open question. We only apply mathematical techniques to convert the two models from the literature into *T_c_* values, which may be important to find out the possible mechanism of iron-based superconductors.

## 2. Computational Methods

As a starting point, the electronic properties of all compounds investigated in this article are computed by the spin-unrestricted Generalized Gradient Approximation of the Perdew–Burke and Ernzerhof (GGA-PBE) functional (unless otherwise specified) [31,32,33,34,35] in Wien2K. The SCF tolerance is 1 × 10^−5^ eV, and the interval of the k-space is 0.025(1/Å). The maximum SCF cycle is 1000. The magnetism and phonon data are calculated by CASTEP. Finite displacement mode is chosen where the supercell defined by cutoff radius is 5 Å and the interval of the dispersion is 0.04(1/Å). Ultrasoft pseudopotential is assigned, and density mixing is chosen to be the electronic minimizer [31,32,33,34,35]. The experimental lattice parameters are used [36,37]. In this article, only Fe and As atoms are imported for the 111-type compounds.

Instead of calibrating “A” in the GGA+A functional, which entails an enormous computational cost and time-consuming experimental effort [21,38,39], we propose a two-channel model to more easily model the induced *xy* potential, where the upper tetrahedral plane is called channel 1 and the lower tetrahedral plane is called channel 2, respectively. We apply the superposition principle to separately calculate the induced *xy* potentials induced by channels 1 and 2. Our two-channel model has fulfilled an assumption that the probability of finding an Fe atom moving in the +*z* and −*z* directions is equal, but their vibrational amplitudes never cancel each other out. This assumption is justified by Coh et al., whose explicit calculation confirms that the iron-based system consists of an out-of-phase vertical displacement of iron atoms, with the first adjacent iron atoms moving in opposite directions [21]. We define $R_{ph}=\frac{0.5\left(DOS_{1}^{XY}+DOS_{2}^{XY}\right)}{DOS_{12}^{XY}}$. In the ARPES range, $DOS_{1}^{XY}$ represents the average electronic density of states for the structure that exclusively contains upper tetrahedral planes. Similarly, $DOS_{2}^{XY}$ indicates the average electronic density of states within the ARPES range for the structure that only contains lower tetrahedral planes. Meanwhile, $DOS_{12}^{XY}$ corresponds to the average electronic density of states within the ARPES range for the original structure that has coexisting upper and lower tetrahedral planes.

F(ω) is the phonon density of states as a function of frequency ω, and the integral ${\int d^{2}p}_{F}$ is taken over by the Fermi surface with the Fermi velocity $v_{F}$. The Eliashberg function is written as [40]

$$\alpha_{}^{2}F(\omega )=\int \frac{d^{2}p_{F}}{v_{F}}\int \frac{d^{2}{p_{F}}^{\prime }}{{\left(2\pi \hbar \right)}^{3}{v_{F}^{}}^{\prime }}\sum_{v}g_{pp^{\prime }v}^{2}\delta \left(\omega -\omega_{p-p^{\prime }v}^{}\right)/\int \frac{d^{2}p_{F}}{v_{F}}$$

The electron–phonon matrix elements are given by $g_{pp^{\prime }v}=\sqrt{\frac{\hbar }{C\omega_{p-p^{\prime }v}}}g_{v}(p,p^{\prime })$, where $\int \psi_{p}^{*}u_{i}\cdot \nabla V_{XY}\psi_{p\prime }^{}dr$ is abbreviated as $g_{v}(p,p^{\prime })$, $\psi_{p}^{}$ is the wavefunction of electron, ℏ is the Planck constant divided by 2π and C is the material constant related to lattice [40]. $u_{i}$ and $V_{XY}$ represent the displacement of the ion relative to its equilibrium position and the ionic potential. $\psi_{p}^{*}\psi_{p}^{}$ is the electronic probability density in the nonmagnetic state. The resultant ionic interaction $V_{ion}^{XY}$ on the *XY* plane, due to the abnormal phonon, is calculated by multiplying the ionic potential by $R_{ph}$, i.e., $V_{ion}^{XY}=V_{XY}\cdot R_{ph}$. Moreover, the antiferromagnetic interaction along the *XY* plane modifies the electronic wavefunction $\phi_{p}^{}$, and the probability density fulfills $\phi_{p}^{*}\phi_{p}^{}\sim \psi_{p}^{*}R_{SDW}\psi_{p}^{}$. The spin density wave factor $R_{SDW}^{2}$ can be considered as the amplification factor for electron–phonon scattering under an antiferromagnetic SDW state, relative to a nonmagnetic state [21]. Rearranging the mathematical terms yields the electron–phonon matrix element as

$$g_{pp^{\prime }v}=\sqrt{\frac{\hbar }{C\omega_{p-p^{\prime }v}}}\int u_{i}\cdot \nabla \left(V_{XY}R_{ph}\right)\psi_{p}^{*}R_{SDW}\psi_{p^{\prime }}^{}dr=\sqrt{\frac{\hbar }{C\omega_{p-p^{\prime }v}}}\int \phi_{p}^{*}u_{i}\cdot \nabla V_{ion}^{XY}\phi_{p}^{}dr$$

To derive a superconducting transition temperature from the simulation parameters, we use the McMillan *T_c_* formula [40]. Due to the high transition temperatures, the electron–phonon scattering matrix takes into account the full electronic DOS in a range from $E_{F}-E_{Debye}$ to $E_{F}$ and not only the value at Fermi level (i.e., increasing the effective electronic DOS). Here, we consider that $E_{Debye}$ represents the upper limit of the phonon energies that can be transferred to electrons, and at the high transition temperatures of Fe-based superconductors, contributions from high-energy phonons become important in the electron–phonon scattering mechanism, as opposed to classical low-*T_c_* superconductors. Although this approach is a simple consequence of the conservation of energy, it is supported by experiments: a shift of the spectral weight between the normal and the superconducting state is clearly visible in the photoemission spectra below the superconducting energy gap of various iron-based compounds in an energy range of ~30–60 meV below the Fermi energy [18,19,20]. This energy range is approximately on the order of Debye energy.

In Bardeen–Cooper–Schrieffer (BCS) superconductors, the electrons on the Fermi surface condense into the Bose–Einstein superconducting state, where the total number of electrons on the Fermi surface equals the total number of electrons on the superconducting state. Hence, the theoretical *T_c_* of BCS superconductors remains the same if we substitute either the electronic DOS on the Fermi level or the electronic DOS of the condensed Bose–Einstein state. However, the situation is different in iron-based superconductivity, where the electrons located between $E_{F}-E_{Debye}$ and $E_{F}$ transfer energy to the electrons in the Bose–Einstein superconducting states. When this happens, we have to revise the resultant electron–phonon scattering matrix in the condensed Bose–Einstein state. The Bose–Einstein statistic favors more electrons occupying the superconducting state. The electrons within the ARPES range increases the effective electronic DOS in the condensed Bose–Einstein state indirectly. The electrons within the ARPES range cannot be excited to the Fermi surface due to electrostatic repulsion. However, these electrons have another route to follow the Bose–Einstein distribution, which can be argued as a reason why these electrons disappear below the Fermi level. 

The computation of band structure produces discrete (*E*,*k*) points, where *E* and *k* are the energy and the wavevector of the electron, respectively. The ratio of the electron–phonon scattering matrix is $R_{g}=\frac{\sum_{-\infty }^{E_{F}}g\left(E\right)\delta_{A}\left(E\right)/\sum_{-\infty }^{E_{F}}\delta_{counter}\left(E\right)}{\sum_{-\infty }^{E_{F}}g\left(E\right)\delta_{B}\left(E\right)/\sum_{-\infty }^{E_{F}}\delta_{counter}\left(E\right)}$, which is abbreviated as the ARPES factor. $\delta_{A}\left(E\right)$ is 1 if $\left(E_{F}-E_{D}\right)\leq E\leq E_{F}$. Similarly, $\delta_{B}\left(E\right)=1$ if $E=E_{F}$. Otherwise, $\delta_{A}\left(E\right)=\delta_{B}\left(E\right)=0$. $\sum_{-\infty }^{E_{F}}\delta_{counter}\left(E\right)$ gives the total number of (*E*, *k*) points in the range $-\infty \leq E\leq E_{F}$. $\sum_{-\infty }^{E_{F}}\delta_{A}\left(E\right)/\sum_{-\infty }^{E_{F}}\delta_{counter}\left(E\right)$ or $\sum_{-\infty }^{E_{F}}\delta_{B}\left(E\right)/\sum_{-\infty }^{E_{F}}\delta_{counter}\left(E\right)$ is the percentage of electrons contributed to the $R_{g}$ term. To make a fair comparison, the intervals of k space in the numerator and denominator of $R_{g}$ are essentially the same. The $R_{g}$ term controls the proportion of electrons scattered below the Fermi level. 

Due to the fact that the superconducting transition temperatures are low, we calculate the mean occupation number f(E) in the Fermi–Dirac statistic at low temperatures (T < 100 K), where f(E) and $f(E_{F}-E_{Debye})$ are 0.5 and ~0.5005, respectively. If $DOS(E_{F})/DOS(E_{F}-E_{Debye})\sim 1$, $f(E_{F})/f(E_{F}-E_{Debye})\sim 1$ and $E_{F}>>E_{Debye}$, the tiny offset in the mean occupation number may allow the Eliashberg function to approximately obey the following form.

$$\alpha_{PS}^{2}F(\omega )\sim \langle \sum_{V_{F}-V_{Debye}}^{V_{F}}\int \frac{d^{2}p_{E}}{v_{E}}\rangle \langle \sum_{V_{F}-V_{Debye}}^{V_{F}}\int \frac{d^{2}{p_{E}}^{\prime }}{{\left(2\pi \hbar \right)}^{3}{v_{E}^{}}^{\prime }}\rangle \sum_{v}\delta \left(\omega -\omega_{p-p^{\prime }v}^{}\right){\left|\sqrt{\frac{\hbar }{C\omega_{p-p^{\prime }v}}}\int u_{i}\cdot \nabla \left(V_{XY}R_{ph}\right)\psi_{p}^{*}R_{SDW}R_{g}\psi_{p^{\prime }}^{}dr\right|}^{2}/\langle \sum_{V_{F}-V_{Debye}}^{V_{F}}\int \frac{d^{2}p_{E}}{v_{E}}\rangle$$

where $v_{E}\in (v_{F}-v_{Debye},v_{F})$, and the velocity $v_{Debye}$ is converted from the Debye energy. $\sum_{V_{F}-V_{Debye}}^{V_{F}}\int \frac{d^{2}p_{E}}{v_{E}}$ is the sum of the surface integral $\int \frac{d^{2}p_{E}}{v_{E}}$ at different electron energies within the ARPES range. The form of the antiferromagnetically amplified electron–phonon coupling is expressed as $\lambda_{PS}^{Coh}\sim 2\int \alpha_{PS}^{2}\frac{F\left(\omega \right)}{\omega }d\omega$, where $\alpha_{PS}^{2}\sim \alpha_{E_{F}}^{2}R_{{}_{Ph}}^{2}R_{{}_{SDW}}^{2}R_{g}^{2}$. The $\alpha_{E_{F}}$ is the average square of the electron–phonon scattering matrix on the Fermi surface [40]. In the case of strong coupling, the renormalized electron–phonon coupling is expressed as λPSCoh*=λPSCohλPSCoh+1 [41]. 

When all the terms in the pairing strength at any pressure are entirely calculated by the spin-unrestricted GGA-PBE functional [33], this approach is defined as a “traditional combination of DFT functional”, in which $R_{{}_{SDW}}^{2}$ may be neglected, as the effect of SDW should be included in $\alpha_{E_{F}}^{2}$, $R_{{}_{Ph}}^{2}$, $R_{g}^{2}$ and $F\left(\omega \right)$ automatically in the spin-unrestricted mode. On the other hand, we propose an “empirical combination of DFT functional” which imposes the antiferromagnetic effect on the pairing strength separately. In this case, the antiferromagnetically amplified pairing strength is separately calculated by multiplying the nonmagnetic pairing strength with the antiferromagnetic factors. $\alpha_{E_{F}}^{2}$ and $F\left(\omega \right)$ are computed by spin-restricted mode, but $R_{SDW}^{2}$ always needs an operation of the spin-unrestricted mode in order to add the effect of SDW. As the two-channel model has already mimicked the contributions of the abnormal phonon under antiferromagnetism manually, it is recommended to apply the spin-restricted mode to calculate $R_{{}_{Ph}}^{2}$. Otherwise, the effect of antiferromagnetism on the abnormal phonon may be overestimated. 

For the “empirical combination of DFT functional”, the pairing strength is further corrected by the AF Ising Hamiltonian in the presence of pressure. To include the magnetic effect, this AF Ising Hamiltonian is acquired by the spin-unrestricted GGA-PW91 functional. The pairing strength formulas of LiFeAs (111-type), NaFeAs (111-type) and FeSe (11-type) under pressure are given as λ11111=λPSCoh*f11111(Eex), where $f_{11}^{111}(E_{ex})\sim \frac{{\left[M_{Fe}M_{Fe}E_{co}\right]}_{P>0}}{{\left[M_{Fe}M_{Fe}E_{co}\right]}_{P=0}}$. The ratio $f_{11}^{111}(E_{ex})$ monitors the pressure dependence of the AF energy at each external pressure *P*, and $E_{co}$ is the exchange–correlation energy. We use $f_{11}^{111}(E_{ex})$ to correct the antiferromagnetism under pressure instead of recalculating the $R_{{}_{SDW}}^{2}$. The Debye temperature of the FeSe/SrTiO_3_ is replaced by the vibrational energy of the F-K phonon across the interface [31]. The pairing strength is substituted into the McMillian *T*_c_ formula [27], which includes the enhanced electron–phonon scattering matrix elements: $T_{c}=\frac{T_{Debye}}{1.45}\exp\left(\frac{-1.04\left(1+\lambda_{11}^{111}\right)}{\lambda_{11}^{111}-\mu^{*}\left(1+0.62\lambda_{11}^{111}\right)}\right)$.

## 3. Results

The atomic spring constants between the FeFe bond *k*_FeFe_ and FeSe bond *k*_FeSe_ in the iron-based superconductors are compared. Our DFT calculation shows that *k*_FeSe_/*k*_FeFe_ is ~0.25, while the *k*_FeAs_ is almost 2 times stronger than *k*_FeSe_. As the atomic spring constants of the tetrahedral bonds are comparable to the FeFe bond, the orthogonal phonon appearing is feasible. Our two-channel model demonstrates that the induced *xy* potential is good enough to be emerged at the “GGA-PBE” level. We calculated that the electron–phonon scattering matrix of FeSe under the induced *xy* potential amplified by *R_ph_* = 2.8. While the accuracy of our two-channel model is comparable to the *R_ph_* = 2.2 obtained from the calibrated GGA + A functional [21], we determine the *R_ph_* of NaFeAs and LiFeAs to be 1.97 and 1.8, respectively. The pressure dependence on *R_ph_* is less than ~5% due to *c* >> *a*.

**Figure 1:**
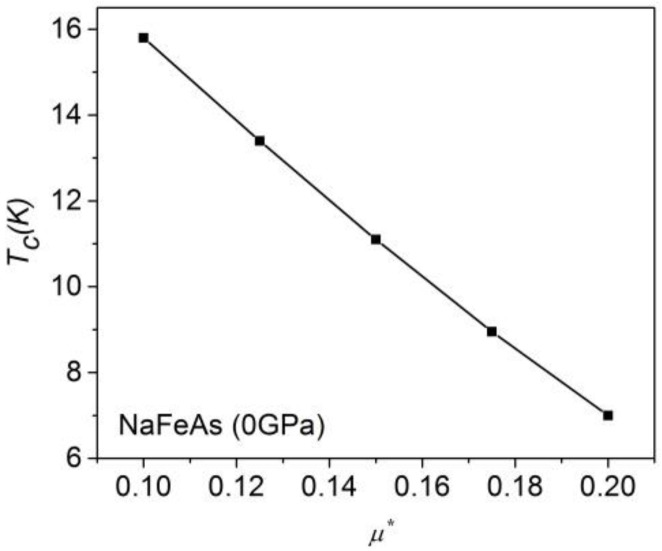
The theoretical *T_c_* of NaFeAs varies slightly with the Coulomb pseudopotential. Our calculated µ*-value of the uncompressed NaFeAs is 0.13.

A critical parameter in any ab initio approach is the value of the renormalized Coulomb pseudopotential. Figure 1 estimates the error of the theoretical *T*_c_ by tuning μ*. Despite that the calculation of μ* as a function of Debye temperature and Fermi level [41] may not be very accurate in such a strongly correlated electron system [42], it has been argued that for the most Fe-based superconductors, μ* should be 0.15–0.2 [12]. In this paper, we choose the value (µ* = 0.15) of the Coulomb pseudopotential to calculate the *T*_c_ of LiFeAs, NaFeAs and FeSe to make a fair comparison. Our calculated µ* value of the uncompressed NaFeAs is 0.13. The error of our *T*_c_ calculation due to the uncertainty of µ* between µ* = 0.15 and µ* = 0.13 is within ~15%.

**Figure 2:**
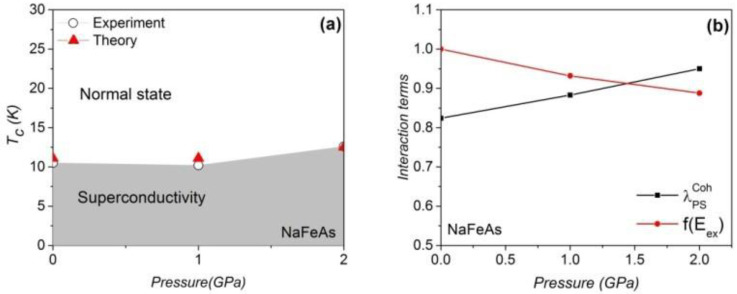
(**a**) The theoretical and experimental [44] *T_c_* values of NaFeAs. (**b**) The antiferromagnetically assisted electron–phonon coupling on the Fermi surface and the AF energy as a function of pressure. The DFT parameter can be found in Table 1.

Figure 2a shows that our approach can generate the theoretical *T*_c_ values in an appropriate range. The ARPES data confirm that LiFeAs and FeSe require the use of the *R*_g_ term, while the NaFeAs does not [18,20,43]. The theoretical *T*_c_ of NaFeAs at 0 GPa and 2 GPa are 11 K and 12.5 K, respectively [44]. The antiferromagnetically enhanced electron–phonon interaction on the Fermi surface and the AF exchange Hamiltonian compete in the compressed NaFeAs, as illustrated in Figure 2b. We observe that the antiferromagnetism is slightly weaker at finite pressure, but the antiferromagnetically assisted electron–phonon coupling on the Fermi layer is increased almost linearly at low pressure. We show the steps to estimate the *T*_c_ of NaFeAs at 0 GPa as an example. After activating the spin-unrestricted mode, the $R_{SDW}^{2}$ is 1.625. The antiferromagnetically assisted electron–phonon coupling on the Fermi surface is


$$\lambda_{PS}^{Coh}=\lambda_{E_{F}}^{}R_{SDW}^{2}R_{ph}^{2}R_{g}^{2}=\left(0.13\right)\left(1.625\right)\left(1.97^{2}\right)\left(1^{2}\right)=0.819,\;\mathrm{and}\;\mu^{*}=0.15.$$

According to the McMillian *T*_c_ Formula, the *T*_c_ becomes

$$T_{c}=\frac{T_{Debye}}{1.45}\exp\left(\frac{-1.04\left(1+\lambda_{11}^{111}\right)}{\lambda_{11}^{111}-\mu^{*}\left(1+0.62\lambda_{11}^{111}\right)}\right)=\frac{385}{1.45}\exp\left(-3.19\right)=10.9K$$

We compare our theoretical *T*_c_ by substituting the raw data of other groups [15,21]; their calculated $\lambda_{E_{F}}^{AF}$ is 0.39 [15], and the induced *xy* potential by the out-of-plane phonon reinforces the electron–phonon coupling matrix by 2.2 [21].

$$\lambda_{PS}^{Coh}=\lambda_{E_{F}}^{AF}R_{ph}^{2}R_{g}^{2}=\left(0.39\right)\left(2.2^{2}\right)\left(1^{2}\right)=1.88$$

After renormalization, these two couplings are softened to λ11111=λPSCoh*=1.88/1.88+1=0.652, and the renormalized Coulomb pseudopotential $\mu_{re}^{*}=\frac{\mu^{*}}{1+\lambda_{PS}^{Coh}}=0.15/\left(1.88+1\right)=0.052$.

Based on the data in other groups [15,21], the theoretical *T*_c_ becomes

$$T_{c}=\frac{T_{Debye}}{1.45}\exp\left(\frac{-1.04\left(1+\lambda_{11}^{111}\right)}{\lambda_{11}^{111}-\mu_{re}^{*}\left(1+0.62\lambda_{11}^{111}\right)}\right)=\frac{385}{1.45}\exp\left(-2.97\right)=13.6K$$

**Figure 3:**
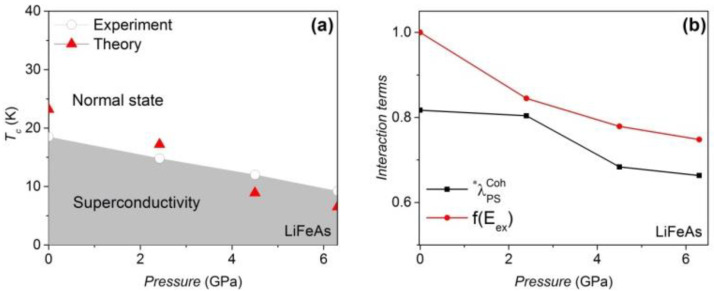
(**a**) The theoretical and experimental [47] *T_c_* values of LiFeAs are consistent. (**b**) The antiferromagnetically assisted electron–phonon coupling and the AF exchange Hamilton under pressure. $R_{SDW}^{2}$ equals 1.75 at 0 GPa.

**Figure 4:**
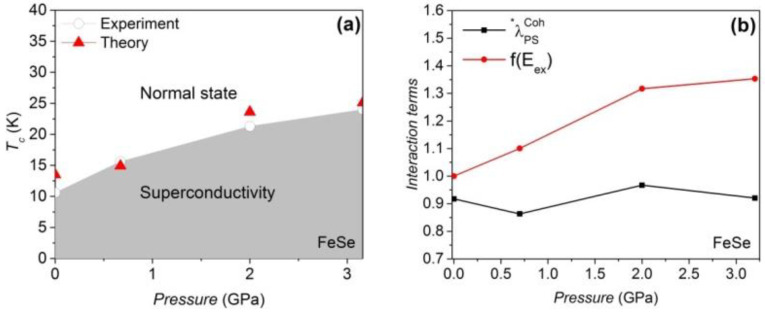
(**a**) Both theoretical and experimental [24] *T_c_* values increase with pressure. (**b**) The pressure dependence of the antiferromagnetically assisted electron–phonon coupling and the AF interaction. $R_{SDW}^{2}$ at 0 GPa is 1.59.

Our calculated value of the electron–phonon coupling on the Fermi surface of the uncompressed LiFeAs is ~0.1 [45], but the magnetic amplification factors increase the pairing strength to 0.82, remarkably. The Debye temperature $T_{Debye}$ of LiFeAs remains at ~385 K below 8 GPa [46], as shown in Table 2. A reduction in the theoretical *T*_c_ is also observed in the compressed LiFeAs, and the weakening effect of λPSCoh* and $f_{11}^{111}(E_{ex})$ under pressure is identified, as shown in Figure 3b. In compressed FeSe [24], however, a gain in $f_{11}^{111}(E_{ex})$ is observed that triggers the increase in *T*_c_ under pressure (Figure 4). It should be noted that our approach is a mean field approach, and we treat the spin fluctuations as being proportional to the mean field Hamiltonian. The vanishing of the macroscopic AF order observed in real samples is due to the strong fluctuation effects in these layered compounds. The magnetism considered here in the nonmagnetic regimes of the phase diagrams is of a fluctuating microscopic nature. The optimized pairing strength of LiFeAs and FeSe is achieved at a pressure of 0 GPa and 0.7 GPa, respectively. The differences between *DOS(E_F_–E_Debye_)* and *DOS*(*E*_F_) in LiFeAs and FeSe are less than 4%. The *R*_g_ term in LiFeAs is reduced with pressure, but the *R*_g_ term of FeSe is optimized at medium pressure (see Table 2 and Table 3).

**Table 1:** The DFT parameter of NaFeAs [46]. The *R_g_* term is computed by the “empirical combination of DFT functional”.

*P*/GPa	*a* (Å)	*c* (Å)	*FeAs Length* (Å)	*R_g_*	*T_Debye_* (K)
0	3.929	6.890	2.400	1.00	385.0
1	3.914	6.833	2.388	1.00	385.5
2.0	3.900	6.777	2.376	1.00	386.0

**Table 2:** The DFT parameter of LiFeAs [46]. The *R_g_* term is compiled by the “empirical combination of DFT functional”.

*P*/GPa	*a* (Å)	*c* (Å)	*FeAs Length* (Å)	*R_g_*	*T_Debye_* (K)
0	3.769	6.306	2.44	2.66	385.00
2.4	3.745	6.134	2.42	2.38	385.25
4.5	3.723	5.985	2.35	1.67	385.5
6.3	3.702	5.918	2.33	1.56	385.75

**Table 3:** The DFT parameter of FeSe [48,49]. The *R_g_* term is simulated by the “empirical combination of DFT functional”.

*P*/GPa	*a* (Å)	*c* (Å)	*FeSe Length* (Å)	*R_g_*	*T_Debye_* (K)
0	3.767	5.485	2.390	3.04	240
0.7	3.746	5.269	2.388	2.05	256
2.0	3.715	5.171	2.384	4.92	274
3.1	3.698	5.114	2.382	2.50	290

**Figure 5:**
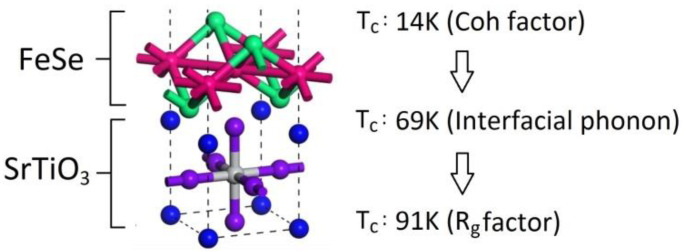
The local region of the unit cell (11 atoms per unit cell). A FeSe monolayered film is deposited on a SrTiO_3_ monolayer to form a composite. The vacuum distance D above the composite is ~52 Å. Our theoretical *T*_c_ values are shown after the amplifications of interfacial F-K phonon, Coh factor and ARPES (or *R_g_* factor) [21,26,31].

Based on the successful *T_c_* calculation of the bulk FeSe, LiFeAs and NaFeAs, we start our journey to acquire the theoretical *T*_c_ of the FeSe monolayer on a SrTiO_3_ substrate step by step using the model of an antiferromagnetically enhanced electron–phonon coupling. The flowchart is shown in Figure 5. After the geometric relaxation of FeSe/SrTiO_3_, the angles of the unit cell are 89.81°, 90.88° and 89.05°, with a tiny internal shear force being captured. The relaxed tetrahedral angle of Fe–Se–Fe is 108 degrees. The antiferromagnetic energy of FeSe can be amplified by low dimensionality when it is deposited in the form of a monolayer on SrTiO_3_ [26]. Compared with a FeSe monolayer without substrate, the FeSe film on SrTiO_3_ shows an increased exchange correlation energy of ~16% on FeSe. Apart from this, the local Fe moment in the isolated FeSe monolayer is only ~0.5 µ_B_. However, contact with SrTiO_3_ amplifies the local Fe moment of the FeSe film up to ~1.3 µ_B_. Our calculated electron–phonon coupling on the Fermi surface without any amplification factor is $\lambda_{Fermi}=0.12$. Based on our simulation, the antiferromagnetism of FeSe/SrTiO_3_ is still as strong as of the FeSe monolayer without substrate. Hence, the simultaneous occurrence of antiferromagnetism and tetrahedral atoms makes the Coh factor unavoidable. The analytical result of C_AF_ = 2 is used [21], and our calculated C_Ph_ in FeSe/SrTiO_3_ is 2.9. After amplification of the Coh factor, the theoretical *T*_c_ is only 14 K. However, a massive enhancement of the pairing strength can be observed when the interfacial F-K phonon is involved [31]. The F-K phonon actuated via the interface contributes the vibrational energy of ~100 meV (~1159 K) [31]. With this enormous Debye temperature, the theoretical *T*_c_ is increased to 69 K, although the electron–phonon interaction is limited to the Fermi energy. In the ARPES data, it is evident that a shift in spectral weight occurs in the superconducting state 0.1~0.3 eV below the Fermi level [19], which means that electrons in this energy range are affected by electron–phonon scattering as a result of the high phonon frequencies. This means that electrons in this energy range contribute to superconductivity, since the high phonon frequencies can scatter them up to the Fermi energy and need to be considered in the McMillan formula, and not only those at the Fermi energy, as in the usual approximation applied to classical low-*T*_c_ superconductors. The superconducting electron concentration is thus corrected, and the average electron–phonon scattering matrix in these multienergy layers is 1.96 times higher than the matrix considering only the Fermi level. This is the last factor with which our theoretical *T*_c_ can reach 91 K, which corresponds quite well to the experimental *T*_c_ of 100 K.

The pairing strength is renormalized as

λPS*=λPSλPS+1=Rg2CF2λFermiRg2CF2λFermi+1=1.962222.9920.121.962222.9920.12+1=0.942

The pseudopotential is diluted as

$$\mu^{*}=\frac{\mu }{1+\lambda_{PS}}=\frac{0.15}{1+\left(1.96^{2}\right)\left(2^{2}\right)\left(2.99^{2}\right)\left(0.12\right)}=0.0085$$

We substitute all parameters into the McMillian *T*_c_ formula:

Tc=TDebye1.45exp−1.041+λPS*λPS*−μ*1+0.62λPS*

$$=\frac{1159}{1.45}\exp\left(\frac{-1.04\left(1+0.942\right)}{0.942-0.0085\left(1+0.62\left(0.942\right)\right)}\right)=91K$$

## 4. Discussion

### 4.1. Are ARPES Data and the Coh Factors Important to IBSC?

The pure FeAs layer in the 111-type, 1111-type and 122-type Fe-based superconductors is believed to trigger superconductivity [50,51]. The investigation of the pure FeAs layer without the Li and Na atoms in the simulation can show the bare pairing strength. The *T*_c_ vs. pressure of the NaFeAs is not as sensitive as for the other materials. The reason for this is that the increase in λPSCoh* and the decrease in $f_{11}^{111}(E_{ex})$ almost cancel out the variation in the pairing strength. The unusually high *T*_c_ in the LiFeAs and FeSe at 0 GPa is mainly due to the $R_{ph}$, $R_{SDW}$ and $R_{g}$ terms (Coh factor: $R_{ph}$ and $R_{SDW}$; ARPES factor: $R_{g}$). Our approach confirms that the reduction in *T*_c_ in compressed LiFeAs is mainly due to the decreases in λPSCoh* and AF energy as a function of pressure. Conversely, the magnetic moment of Fe in FeSe increases under compression, resulting in an increase in AF energy under pressure. As a result, the increase in *T*_c_ in compressed FeSe is observed. The *R*_g_ term is minimized at high pressure, since the kinematics of electrons below the Fermi level is more restricted under pressure. Our simulation shows that the variation in the induced *xy* potential is less than ~3% for the electrons at ~100 meV below the Femi level, and therefore, the use of the APRES factor (or *R*_g_) in LiFeAs and FeSe is justified.

We correct the pairing strength at high pressures with the help of the AF Ising Hamitonian. In the following, we compare the *T*_c_ when $R_{g}^{}$ and $R_{ph}^{}$ are calculated by the spin-unrestricted GGA-PBE functional at high pressures, simply called the “traditional combination of DFT functional”. Despite the “traditional combination of DFT functional” providing an accurate theoretical *T*_c_ at ambient pressure, the error of *T*_c_ is significant at high pressures. We demonstrate this for the case of FeSe in Table 4. In this approach, we do not use the AF Ising Hamiltonian at finite pressure because magnetism is already considered. Since 2008, the ARPES factor (*R*_g_) has been missing in the calculation of the electron–phonon coupling constant. However, Table 5 confirms that the consideration of the electron–phonon coupling on the Fermi surface is not sufficient to argue whether iron-based superconductivity is mediated by phonons. If the ARPES factor (*R*_g_) really participates in iron-based superconductivity, the abnormal distribution of electrons below the Fermi level should be given a larger range when the *T*_c_ of the iron-based superconductor is higher. This argument is supported by the ARPES data of the 100 K 2D FeSe/SrTiO_3_ [19] with the parameters shown in Table 6. For these ~10–30 K iron-based superconductors, the electrons located at 0.03–0.06 eV below the Fermi level are affected by superconductivity [22,29]. However, the electrons in the 100 K 2D FeSe/SrTiO_3_, which are located in a much wider range of 0.1–0.3 eV below the Fermi level, participate in superconductivity [19]. The theoretical *T*_c_ of the 2D FeSe/SrTiO_3_ reaches 91 K only if the ARPES factor (*R*_g_) is considered.

An empirical rule is that the *T*_c_ of the iron-based superconductor is optimized when the tetrahedral angle is close to 109.5 degree [52]. When the FeSe monolayer is attached to the SrTiO_3_, the tetrahedral angle is changed from 103 degrees to 108 degrees, and *T*_c_ benefits. However, all these antiferromagnetic and tetrahedral effects cannot explain the high *T*_c_ near 100 K until the interface properties are considered [31]. Despite the Debye temperature of the FeSe phonons (~250 K) showing no significant size effect, an energetic F-K phonon carrying an energy of 100 meV (~1159 K) was observed at the interface between the FeSe film and SrTiO_3_ [31]. Since the 3D and 2D FeSe phonon are almost identical [31], the out-of-plane phonon from the tetrahedral sites should amplify the electron–phonon coupling of FeSe/SrTiO_3_ by the same *R_Ph_* factor = 2. Assuming that the F-K phonon and FeSe phonon interact with electrons simultaneously, two Debye energies, i.e., from the FeSe phonons and the F-K phonons, may influence the Cooper pairs. The two-fluid model, however, ensures that the onset *T*_c_ is always related to the mechanism that gives the strongest pairing strength, and therefore, choosing 1159 K as the Debye temperature is justified.

The ARPES data of FeSe/SrTiO_3_ show that the electrons in a wide range below the Fermi level (Δ*E*~0.1–0.3 eV) participate in superconductivity [19]. A question may be asked: Which energy source causes this shift of spectral weight? The F-K phonon may be one of the options since the *E_Debye_* is ~0.1 eV [31]. Would it be exchange coupling? The exchange–correlation energy *E*_co_ of FeSe/SrTiO_3_ is also ~0.1–0.2 eV. However, we believe that the F-K phonon is the energy source to generate this shift of spectral weight in FeSe/SrTiO_3_. To support our argument, we revisit the ARPES results [18,20], where the bulk iron-based superconductors carrying *E_co_*~0.1 eV displayed a shift in spectral weight at Δ*E*~30–60 meV below the Fermi level. If the shift is caused by the exchange–correlation energy, Δ*E* and *E_co_* should be comparable in the bulk iron-based superconductors, but this is not the case. If the exchange correlation energy is not the correct answer, we reinvestigate the magnitude of *E_Debye_*. Interestingly, the narrower range Δ*E*~30–60 meV is comparable to the Debye temperature [53,54] of bulk iron-based superconductors. With this, we believe that Δ*E*~*E_Debye_* is unlikely to be a coincidence. The shift of spectral weight in ARPES in iron-based superconductors is thus likely triggered by phonon-mediated processes. After revising the electron concentration in the superconducting state, our calculated *T*_c_ is further increased to 91 K. We verified that the Coh factor is only reduced by ~3% at E_F_-100 meV.

### 4.2. Would the Errors in T_c_ Be Rescued by Nematicity and Spin–Orbital Coupling?

On the Fermi surface, a nematic order may be observed in various iron-based superconductors [52,55], and the electron–electron interaction should be influenced accordingly. Although our approach does not consider the nematic order, our approach averages the electron–phonon coupling between *E_F_*–*E_Debye_* and *E_F_*, which pales the contribution from the nematic order on the Fermi surface. The numerator of $R_{g}$ contains the average electron–phonon scattering matrix in multienergy layers, where the Fermi energy is only one of them. Under these circumstances, the error of $\alpha_{PS}$ from neglecting the nematic effect may be relatively small (the variation of *T_c_* enhanced by nematic phase in S-doped FeSe is just a few kelvins! If the nematic phase is encountered in our approach, this may help increase the calculated *T_c_* to 100 K; however, the *T_c_* calculation based on the concept of the nematic phase is still an open question), and our *T*_c_ calculation should remain accurate. The spin–orbital coupling SO may be a reason for triggering the unusually high *T_c_* in FeSe/SrTiO_3_ due to the heavy elements in SrTiO_3_ [56,57]. If the effect of SO is taken into account, the calculated *T_c_* may move even closer to the experimental value. Additionally, another source of error in the *T_c_* of FeSe/SrTiO_3_ may be caused by the thickness of SrTiO_3_ used in the simulation. The theoretical *T_c_* of FeSe may increase as the thickness of SrTiO_3_ is increased in the simulation in the future.

### 4.3. The Universal Theory of IBSC Remains an Open Question

The *T*_c_ acquired by the “traditional combination of DFT functional” fails at high pressures, mainly because $R_{g}$ is excessively suppressed. To monitor electron–phonon coupling under pressure, the use of the “empirical combination of DFT functional” is a better choice. Although the accuracy of the GGA-PBE/PW91 functional may not be perfect, we empirically correct the numerical output value $\lambda_{11}^{111}$ directly via the AF Ising Hamiltonian and the two-channel model. On one hand, the two-channel model corrects the effect of the out-of-plane phonon at a low computational cost. On the other hand, the introduction of the induced *xy* potential in the electron–phonon calculation indirectly corrects the effect of the band diagram. The $\lambda_{11}^{111}$ is controlled by the band diagram, which contains the information about the effective mass. The numerator and denominator in *R*_g_ are obtained from the same band diagram, so that the error due to the effective mass in these three nonheavy fermion superconductors can almost be cancelled.

It is still an open question which DFT functional is the best for iron-based superconductors. From an empirical point of view, the one-body Green’s function and the dynamically screened Coulomb interaction (GW), or screened hybrid functional, are likely suitable for unconventional bismuthate and transition metal superconductors [58]. The modeling of the Hubbard potential in the GGA+U approach provides good agreement with the experimental results of BaFe_2_As_2_ and LaFeAsO [38]. Since the electron–electron interaction in the iron-based superconductors is complicated, the use of the highly correlated DFT functional should be reasonable. However, the *T*_c_ calculated with the screened hydrid functional HSE06 convinces us to use a different approach. We calculate the *T*_c_ of these three materials by the HSE06 functional, which is a class of approximations to the exchange–correlation energy functional in density functional theory, which includes a part of the exact exchange item from the Hartree–Fock theory with the rest of the exchange–correlation energy from other sources [38]. However, the exchange–correlation energy considered by the screened hydrid functional HSE06 does not suit the NaFeAs, LiFeAs and FeSe materials, whose calculated *T*_c_ values become less than 0.1 K. The more advanced approaches, such as GW or dynamical mean-field theory (DMFT), can simulate most of the electronic properties of bulk FeSe closer to the experimental values, but the major drawback is that the calculation of the electron–phonon coupling with these methods is based on a simplified deformation potential approximation, since electron–phonon coupling matrix elements are difficult to obtain [39].

The induced *xy* potential was rarely reported at the GGA level. If the channels where the out-of-plane phonon cannot be hidden are considered separately, the GGA functional is already good enough to generate the induced *xy* potential. If the lattice Fe moves orthogonally away from the *xy* plane in the iron-based superconductors, the electric charges in the *xy* plane are disturbed. Since the electronegativity of the tetrahedral atom (Se or As) is stronger, the electron will populate the FeSe or FeAs bonds more [21]. For example, when the Fe moves along the +*z* axis, the local electron density in the *xy* plane changes. The induced charges have two possible paths, i.e., the electrons are shifted either above or below the *xy* plane to the FeSe (or FeAs) bond [21]. However, the upward displacement of the Fe atom, which emits the electric field, confines the electrons more covalently in the upper tetrahedral region. The more covalently bonded FeSe (or FeAs) interaction allows electrons to move out of the FeSe or (FeAs) bond below the plane [21]. A charge fluctuation is created and generates the induced *xy* potential. Since the out-of-plane phonon is simulated by the two-channel model, the occurrence of the induced *xy* potential at the GGA level means that the two-channel model has already taken the AF into account.

The McMillian formula takes into account the distribution of electrons in the form of a hyperbolic tangent (*tanh*) function across the Fermi level [40]. At finite temperature, the Fermi–Dirac statistics fit the shape of the hyperbolic tangent function with the mean occupation number $f(E_{F})=0.5$. For example, elemental aluminum holds the superconducting transition temperature at 1.2 K, where the offset $f(E_{F}-E_{Debye})-f(E_{F}+E_{Debye})$ is 0.0056. In addition, the offset $f(E_{F}-E_{Debye})-f(E_{F}+E_{Debye})$ of elemental tin is 0.0028 at ~3 K. The McMillian formula provides the theoretical *T*_c_ of aluminum and tin correctly with the tiny offsets of 0.0056 and 0.0028, respectively. The relevant electrons in the studied superconductors may be located in the energy range between $E_{F}-E_{Debye}$ and $E_{F}+E_{Debye}$, but their offsets $f(E_{F}-E_{Debye})-f(E_{F}+E_{Debye})$ at low temperatures are as small as ~0.005. If $f(E_{F}-E_{Debye})-f(E_{F}+E_{Debye})$ in the iron-based superconductors are comparable to BCS superconductors, the numerical error due to the fitting of the relevant electrons indicated by the energy range we extracted from ARPES data as input in the McMillian formula and the Eliashberg function may not be obvious. If the APRES factor (*R*_g_) is introduced in a narrow energy range below the Fermi level, it fits even better with the *tanh* function. Furthermore, the AF Ising model shows that the energy of the spin fluctuations is smaller than the Debye energy, and hence, the maximum integral in the McMillian derivation [40] cannot exceed the Debye temperature. Finally, none of the amplified electron–phonon couplings exceed the limit of the straight-line fit for determining the empirical parameters [40]. Therefore, the McMillian formula becomes applicable in these three iron-based superconductors.

After we consider all electrons taking part in iron-based superconductivity between *E*_F_ and *E*_F_–*E*_D_, the calculated *T*_c_ of the above samples are much closer to experimental values. We thus suggest that given the relatively high transition temperatures of Fe-based superconductors at which a considerable amount of high energy phonons are excited, it is absolutely required to consider the entire energy range of electrons that can scatter up to the Fermi energy through these phonons, in contrast to the traditional low-*T*_c_ approaches, where the electronic density of the states at the Fermi level can be used as an approximation. For a proposed theory of iron-based superconductors to be deemed incorrect, an unified theory of iron-based superconductors would need to have already existed. However, what is the unified theory of iron-based superconductor? It is still an open question. Despite our algorithm producing reasonable theoretical *T*_c_ for these four samples, this article only combines several proposed mechanisms of IBSCs instead of presenting a comprehensive theory. However, our research provides optimism for scientists that accurate *T_c_* calculations in iron-based superconductors may be possible. It is crucial to conduct further theoretical work to develop a unified theory of iron-based superconductors that can accurately predict the theoretical *T*_c_ of all iron-based superconductors.

## 5. Conclusions

After revising the superconducting electron concentration in the McMillan *T*_c_ formula, we could show that when the conduction electrons interact with local Fe moments in Fe-based superconductors, the coexistence of superconductivity with local fluctuating antiferromagnetism together with the abnormal lattice vibration, which can lead to an enormous increase in the electron–phonon coupling, is sufficient to predict the high *T*_c_ values. Our ab initio approach can generate theoretical *T*_c_ values of NaFeAs, LiFeAs and FeSe close to the experimental values. When the model is applied to monolayered FeSe on a SrTiO_3_ substrate, we find that the interfacial phonons are of major importance to explain the high-temperature superconductivity.

## Figures and Tables

**Table 4:** The theoretical *T_c_* of FeSe at different pressures. Theoretical *T_c_* (A) is obtained from the traditional combination of DFT functional. Theoretical *T_c_* (B) is estimated from the empirical combination of DFT functional.

FeSe	Experimental *T*_c_	Theoretical *T*_c_ (A)	Theoretical *T*_c_ (B)
0 GPa	11 K	13 K	12 K
0.7 GPa	16 K	4 K	15 K
2 GPa	20 K	3 K	22 K

**Table 5:** Effect of ARPES factor (*R_g_*) on theoretical *T_c_* values. The “empirical combination of DFT functional” is used.

FeSe	Experimental *T*_c_	Theoretical *T*_c_ (Without *R_g_* Term)	Theoretical *T*_c_ (With *R_g_* Term)
0 GPa	11 K	3 K	12 K
0.7 GPa	16 K	6 K	15 K
2 GPa	20 K	8 K	22 K
**LiFeAs**	**Experimental *T*_c_**	**Theoretical *T*_c_ (Without *R_g_* Term)**	**Theoretical *T*_c_** **(With *R_g_* term)**
0 GPa	19 K	2 K	23 K
2.4 GPa	15 K	7 K	17 K
4.5 GPa	13 K	8 K	9 K
6.3 GPa	10 K	4 K	7 K

**Table 6:** The simulation parameters of FeSe/SrTiO_3_ [29]. The unit cell of FeSe/SrTiO_3_ occupied a volume of 3.8197 Å × 3.8698 Å × 5.9540 Å. The vaccum distance D above the composite is 52.484 Å.

*a* (Å)	*b* (Å)	*c* (Å)	*D* (Å)	λ*_Fermi_*	*R_g_*	*Debye* (K)
3.8197	3.8698	5.9540	52.484 Å	1.6	1.96	1159

## Data Availability

Data are sharable under reasonable request. The authors are usually supportive for reproducing the results if further assistance is needed (please send your technical requests to roywch654321@gmail.com).
